# Comment on “Intervention Effects of a School-Based Health Promotion Programme on Obesity Related Behavioural Outcomes”

**DOI:** 10.1155/2015/708181

**Published:** 2015-05-25

**Authors:** Peng Li, Andrew W. Brown, J. Michael Oakes, David B. Allison

**Affiliations:** ^1^Office of Energetics and Nutrition Obesity Research Center, University of Alabama at Birmingham, Birmingham, AL 35294, USA; ^2^Division of Epidemiology and Minnesota Population Center, University of Minnesota, Minneapolis, MN 55454, USA

The paper, “Intervention Effects of a School-Based Health Promotion Programme on Obesity Related Behavioural Outcomes” by Kobel et al. [1], reports secondary outcomes from a cluster-randomized controlled trial (cRCT): the Baden-Württemberg primary school study (DRKS-ID: DRKS00000494) [2]. Importantly, the design of this cRCT properly incorporated crucial aspects of such trials, such as the lack of independence of subjects within clusters and the nesting of clusters within treatment conditions [2]. Additionally, plausible analytical models (e.g., linear mixed effects models or GEE models) were planned [2]. Unfortunately, the statistical analysis ultimately reported in [1] is inconsistent with the predefined analysis plan and does not take the impact of clustering and nesting into account. Ignoring the potential similarity among individuals in the same cluster (school) can underestimate the variance of intervention effects and inflate the degrees of freedom in the hypothesis testing and, therefore, increase the type I error rates and jeopardize the validity of conclusions from cRCTs [3, 4].

We agree with others that the cRCT is one of the most important designs in the community- or school-based obesity studies. Unfortunately, many seem not to understand the implications of clustering and nesting and the need for specialized statistical models to analyze such data. To be consistent with recommended best practices and to improve the transparency and utility of cRCTs, we suggest that Kobel et al. reanalyze their data according to their prespecified plan by taking the clustering and nesting into account and report their results following the CONSORT 2010: extension to cluster-randomized trials [5].
